# Evolutionary ecology meets the antibiotic crisis: Can we control pathogen adaptation through sequential therapy?

**DOI:** 10.1093/emph/eoz008

**Published:** 2019-02-22

**Authors:** Roderich Roemhild, Hinrich Schulenburg

**Affiliations:** 1 Department of Evolutionary Ecology and Genetics, Zoological Institute, Christian-Albrechts-Universität zu Kiel, Am Botanischen Garten 1-9, Kiel, Germany; 2 Antibiotic Resistance Evolution Group, Max-Planck-Institute for Evolutionary Biology, August-Thienemann-Str. 2, Plön, Germany

**Keywords:** sequential therapy, fluctuating selection, antagonistic pleiotropy, collateral sensitivity, negative hysteresis

## Abstract

The spread of antibiotic resistance is a global challenge that is fueled by evolution and ecological processes. Therefore, the design of new sustainable therapy should take account of these underlying processes—as proposed within the field of evolutionary medicine, yet usually not receiving the necessary attention from national and international health agencies. We here put the spotlight on a currently neglected treatment strategy: sequential therapy. Changes among antibiotics generate fluctuating selection conditions that are in general difficult to counter by any organism. We argue that sequential treatment designs can be specifically optimized by exploiting evolutionary trade-offs, for example collateral sensitivity and/or inducible physiological constraints, such as negative hysteresis, where pre-exposure to one antibiotic induces temporary hyper-sensitivity to another antibiotic. Our commentary provides an overview of sequential treatment strategies and outlines steps towards their further optimization.

## THE PROBLEM OF ANTIBIOTIC RESISTANCE EVOLUTION

The rise of antibiotic resistance is a growing challenge for global health. Resistances emerge and spread rapidly as a consequence of antibiotic use in both medical treatment and agriculture, potentially compromising the treatment of infectious disease [1]. The current antibiotic crisis is essentially an evolutionary problem that is determined by selection (the particular antibiotic treatment used), the evolving organisms (bacteria and their population characteristics) and the genetics of adaptation (space of resistance mutations and the distribution of their fitness effects). Surprisingly, the high potential of bacteria for adaptation is usually not part of the design of novel therapy. With this commentary, we therefore explore how concepts based in evolutionary ecology may yield new ideas for sustainable antibiotic therapy. We identify sequential therapy as a highly potent treatment option, which should make it difficult for bacteria to adapt because of the continuously changing selective challenge. In the following, we will illustrate why current procedures of antibiotic treatment are often sub-optimal from the view of evolutionary ecology. We then discuss ecological principles that may improve treatment sustainability by limiting the rate of *de novo* resistance evolution. The discussion is focussed on improving the treatment of chronic infections, as these are prone to resistance evolution by chromosomal mutation. We conclude by outlining research directions towards the clinical implementation of the proposed evolution-informed therapy.

## SUB-OPTIMALITY OF COMMON TREATMENTS

**Figure 1.**
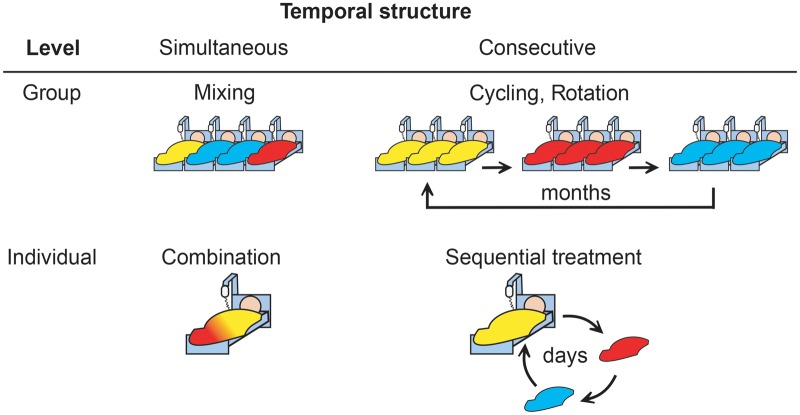
Strategies for multidrug treatments. Multidrug treatments can be designed in different ways, depending on the temporal structure and the application level. Colours represent different drugs

Historically, the first strategy for antibiotic therapy was to treat patients for several days with an antibiotic, typically of broad-range activity, such as penicillin. Such monotherapies are still the main treatment form today, yet resistance to the single drugs can evolve rapidly through natural selection [1]. Fast adaptation to individual antibiotics is usually caused by three main non-exclusive factors: (i) a high number of different mutations can confer resistance and these may easily arise due to usually large bacterial population sizes and/or horizontal gene transfer, (ii) the selective advantage of any resistance mutation is large, even if originally rare, and thus they can spread fast through the population (i.e. growth advantage of the resistant variant over the susceptible variant) and (iii) competitive release and thus the reduction in often detrimental direct interactions with non-resistant competitors can further favour the resistant varieties. Evolutionary biologists seek ways to prevent the rapid fixation of resistance mutations by limiting these processes. One approach is to increase the complexity of the environments by applying several different drugs within a single treatment [2]. It is more likely for bacteria to become resistant to a single drug than to several drugs, because there are fewer mutations that provide cross-resistance (although there are noteworthy exceptions [3]). These drugs can be deployed simultaneously or consecutively and at different hierarchical levels that focus on either patient groups or single individuals (Fig. 1). The approaches have different rationales: group level application (hospital, cohort, intensive care unit) aims at limiting the spread of resistance caused by cross-infection. Application in single patients aims at preventing resistance emergence during treatment.

Simultaneous multidrug treatment of patient groups is termed ‘mixing’ therapy [4]. Within an intensive care unit (ICU) multiple antibiotics are applied on the same day, but patients individually only receive a single drug (Fig. 1). Throughout the whole treatment, medication of a patient remains constant, such that each patient effectively receives monotherapy. This strategy produces a patchy selective environment and thus increases spatial but not temporal variation. Therefore, the likelihood of *de novo* resistance evolution in a single patient is not decreased over monotherapy.

Combinations of two or more drugs within the same patient (Fig. 1) produce more complex adaptive landscapes due to drug interaction. Drug interaction can provide immediate advantage if drugs synergistically enhance their inhibitory effect on bacterial growth. Certain antibiotic combinations have therefore been used to combat infections efficiently and combination treatment is now the standard for several bacterial infections [5, 6]. However, simultaneous drug deployment was repeatedly observed to accelerate evolutionary rescue *in vitro* [7–9]. Resistance evolved earlier in experimental populations treated with combinations than in populations treated with monotherapy, because aggressive treatments release rare multidrug resistant variants from competition with non-resistant cells. The higher initial efficacy of combination treatments is thus offset by faster resistance emergence. This may explain, why clinical trials failed to show a general advantage in patient recovery and survival after combination therapy as compared to monotherapy [10]. Such dynamics may be partially circumvented by special drug combinations that show suppressive interaction [11]. These combinations can limit bacterial resistance evolution by selecting against mono-resistant mutants in a specific concentration window. Yet, because of their suppressive effect upon one another, the total drug concentration of the pair needs to be higher than that required in monotherapy to achieve the same inhibitory effect, potentially causing stronger side-effects for the patient [2].

Sequential drug protocols are an alternative treatment strategy that may unite the benefits of combination therapy with sustainability, due to additional adaptive constraints caused by the temporal complexity. To date, the idea of sequential treatment has been applied mostly on the group level. In ‘rotation’ or ‘cycling’ therapy the whole patient group is treated with the same antibiotic, which is periodically switched for a new antibiotic after several weeks (Fig. 1). As switching interval is longer than hospital stay, the likelihood of resistance emergence is not reduced within an individual compared to monotherapy. A recent meta-analysis of clinical trials for cycling therapy could show an overall benefit compared to mixing [12] but this effect was due to a reduced number of hospital acquired infections and not because selection for resistance was minimized [13]. We argue that sequential therapy can minimize resistance evolution, but not when it is carried out with the current unit-wide approach and the long switching intervals. Drug resistance does evolve within single patients. To limit the emergence of resistance, multidrug treatments should be applied to one patient, such that they potentially affect a single population of the pathogen. Thus, drugs should be alternated rapidly, for example each day (Fig. 1) or even more often. Frequent switching produces fluctuating selection to which adaptation is more difficult. Any particular switch of antibiotics during treatment may improve treatment outcome by curing strains resistant to the preceding antibiotic [12]. Clinical trials on fast sequential treatments proved effective against *Helicobacter pylori* infections [14]. Likewise, sequential therapy increased eradication of *Pseudomonas aeruginosa* in a small cohort of cystic fibrosis patients [15]. Intriguingly, the latter study was already published 30 years ago in the *Lancet*, but did not receive much attention (less than 10 citations since publication according to Web of Science).

## CONTROLLING RESISTANCE EMERGENCE BY TEMPORAL VARIATION

Sequential treatments complicate resistance evolution because they produce dynamically changing adaptive landscapes for pathogen populations. The selection dynamics can be optimized according to eco-evolutionary principles. We argue that the full potential of sequential treatments can be achieved by considering (a) pleiotropic fitness effects of resistance mutations, (b) physiological interactions that occur at switches between drugs, (c) a sufficient rate of environmental change and (d) sequence irregularity.

### Pleiotropic genetic interactions

Antibiotic resistance is a pleiotropic trait that usually entails ecological trade-offs. Most proteins, and particularly antibiotic resistance genes, are part of interconnected biological networks. As a consequence, adaptive mutations nearly always affect the expression of multiple traits (i.e. pleiotropic effects). Adaptive mutations are therefore often associated with fitness trade-offs in distinct environments [16, 17]. In the context of antibiotic treatment, switching drugs in a specific order can potentiate treatment and re-sensitize bacteria due to the antagonistic pleiotropy of previous resistance mutations.

The importance of pleiotropy for the evolution of resistance has recently been emphasized by the rediscovery of the concept of collateral sensitivity, originally introduced more than 60 years ago (Box 1). The evolution of resistance to one antibiotic can increase susceptibility to antibiotics of other classes. The published sensitivity maps [18–22] show antibiotic class specific patterns, which indicates that collateral sensitivity originates from constraints caused by the general ‘Bauplan’, i.e. structural architecture of the cell. Functional genetic analysis confirmed that collateral sensitivity can result from resistance mutations against one drug that simultaneously facilitate uptake of another antibiotic. Such collateral sensitivity was found for strains of *Escherichia coli* adapted to aminoglycoside antibiotics [18, 20, 21]. Resistance against aminoglycosides is often caused by mutations that decrease membrane potential, for example, by targeting the K^+^-ion-transporter TrkH [20, 21, 23]. This reduces the uptake of aminoglycosides [24] but also impedes the efficacy of drug efflux pumps such as AcrAB [21], thereby constraining the cellular removal of other drugs, causing hyper-sensitivity. A similar phenotype is achieved by alternative mechanisms in *P. aeruginosa*. Fluoroquinolone-resistant strains of *P. aeruginosa* frequently show collateral sensitivity to aminoglycosides and β-lactams [19, 22, 25], which is caused by mutations that alter the expression of efflux pumps, e.g. via mutation of *nfxB* [26], the major transcriptional repressor of the multidrug efflux pump MexCD-OprJ [27], or other efflux regulators such as *mexZ* or *nalC* [19]. The resulting changes in expression of particular efflux pumps however affects expression of alternative pumps [27], suggesting that collateral sensitivity is caused in these cases by a deviation from natural efflux balance.

Recent experimental tests of sequential treatments that involve collateral sensitivity highlight their potential application in therapy. Evolved *P. aeruginosa* strains that acquired resistance against the β-lactam piperacillin during treatment could be re-sensitized by switching to ciprofloxacin [25], possibly due to *nfxB*-mediated changes in pump expression. Rapid alternating treatments of *E. coli* with drug pairs involving the antibiotic polymyxin resulted in one-sided adaptation and thus the suppression of resistance emergence to one of the drugs [28]. Although the mechanism is not entirely clear, it is likely associated with collateral sensitivity.

A second, potentially very important case of pleiotropy is the typically reduced growth rate of antibiotic resistant mutants (see Ref. [29] for a review), which can result from sub-optimal metabolic flux. The reduced growth rate of resistant mutants is often called a ‘fitness cost’ because it increases competition with non-resistant types and this clonal interference can decelerate adaptation, especially in environments without or with reduced concentrations of antibiotics [29]. This effect may similarly lead to reduced adaptation rates under fluctuating selection conditions, and thus, its potential to enhance sequential therapy is clearly worth a detailed experimental analysis.

### Hysteresis: physiological interactions from cell memory

Bacteria physiologically respond to stress, as caused by antibiotics, by activating stress-response systems that alter transcription of a large number of genes and thereby improve survival for the current conditions. Because many bacterial proteins are stable, induced responses can be phenotypically inherited [30] and may thereby provide cross-stress protection to new conditions. Intriguingly, there are also cases where the previously experienced stressor decreases survival in new stressful environment, a phenomenon called cross-stress sensitivity. One example is NaCl-induced acid sensitivity in *E. coli,* which is mediated by expression of the porin PhoE [31]. Furthermore, there may be less specific cross-sensitivity caused by a metabolic cost of hysteretic response memory [32] or directly by stress-induced damage. Antibiotics themselves can induce responses that entail fitness disadvantages when drugs are switched in sequential treatments. Again, the ecological phenomenon itself was already studied 50 years ago, but has since received negligible attention: sub-lethal pre-treatments with β-lactam antibiotics potentiate killing by aminoglycoside antibiotics in several species of bacteria [33, 34] (Box 2). This phenomenon is called ‘negative cellular hysteresis’, and describes the long-lasting, but non-genetic increase in the susceptibility to one antibiotic, that can be induced by pre-exposure to another antibiotic. Negative hysteresis is distinct from the post-antibiotic effect [35], where brief antibiotic exposure induces a transient suppression of growth in permissive conditions (environment with decreased amount of the same antibiotic or no antibiotic), as opposed to the increased killing by high concentrations of a different antibiotic. Negative hysteresis can help to eradicate chronic infections, as demonstrated experimentally (Box 2) or indicated by the high efficacy of sequential protocols in the treatment of biofilms [36]. In addition to their immediate therapeutic benefits, negative hysteresis was recently shown to inhibit resistance emergence [37]: sequential treatments with three distinct antibiotics, between which there existed strong negative hysteresis, were able to stabilize susceptibility by shifting the priority of adaptation from resistance towards overcoming the physiological interactions [37]. Bacterial populations from these treatments adapted via previously unknown mechanisms that abolished the effect of negative hysteresis without increasing resistance. These data indicate the potency of long-lasting physiological interactions between antibiotics for sustainable therapy.

### Frequency of change

Fluctuating selection can delay adaptation, because it interrupts selective sweeps. For example, rapid but not slow fluctuation in media quality prevented co-evolution between bacteria and phage [38]. Likewise, switching rate can influence the rate of adaptation to antibiotics. If antibiotics are switched too slowly in a sequential protocol, resistance mutations spread through the population, as in monotherapy. In contrast, more rapid fluctuations, such as switching antibiotics every 12 or 24 h, can limit resistance evolution, as recently demonstrated for *E. coli* [9, 28], *P. aeruginosa* [37, 39] and *Staphylococcus aureus* [40], using experimental evolution. Interestingly, these experiments used sub-lethal antibiotic concentrations and achieved both a deceleration of adaptation and also increased population extinction [37, 39]. The latter is likely explained by the increased occurrence of selection pulses as caused by physiological interactions and genetic trade-offs. This model is consistent with the observed lower within-population phenotypic diversity after fast (every 12 h), compared to slow-switching (every 48 h) sequential therapy [37]. Thus, the frequency of change holds promise not only to decelerate adaptation, but also to reduce phenotypic variation, which otherwise could complicate antibiotic treatment.

### Stochastic changes

Unpredictably occurring environmental disturbances are more difficult to adapt to than regularly occurring selective pressures. According to the hypothesis of environmental adaptive conditioning [41], selection can favour an adjustment of gene expression to regular patterns of stimuli. Correlated environmental factors are a common feature of microbial habitats and several microbes exhibit anticipatory gene regulation [42]. These organisms use trigger molecules in their environment to adjust gene regulation for future challenges. One example is *Vibrio cholerae*, which during the last phase of the infection of the human intestine already induces genes necessary for survival in the aquatic environment outside the host [43]. Anticipation was likewise selected by the fixed sequential contrasts in the human gut. Following transmission, *E. coli* encounters lactose in the proximal and maltose in the distal part of the intestine, 3 h later [41]. In the scramble for nutrients, *E. coli* benefits from up-regulating maltose-metabolizing genes ahead of time (lactose induces expression of the maltose operon), thereby skipping the lag-phase associated with the shift in carbon sources. The anticipatory regulation and its fitness advantage are lost when wildtype *E. coli* were grown in constant lactose environment in the lab, indicating a cost of the anticipation behaviour [41]. A mathematical model predicted the evolution of anticipation under certain conditions: strong temporal correlation of stimuli, short time between stimuli and high benefit of the anticipation [42]. These examples may suggest that predictable patterns in sequential antibiotic therapy are potentially dangerous, because they generate the parameter space for the evolution of anticipation. The ensuing adaptive response may be circumvented by irregular drug orders.

Aside from limiting fitness benefits of anticipation, stochasticity in fluctuations can also directly decelerate adaptation to that factor, as demonstrated with populations of viruses, which were exposed to regularly alternating and randomly changing temperatures [44]. In contrast to the observed fitness increases in regularly alternating environments, unpredictable temperature fluctuations led to a significant decrease of fitness [44]. Similarly, fitness returns of bacteria adapting to randomly fluctuating pH were lower than those attained in regularly alternating sequences of pH [17]. The incorporation of temporal stochasticity in sequential treatment protocols may thus additionally restrict resistance evolution in the long-term and may therefore help to control chronic infections. We expect the decelerating effect of randomness to increase with the total number of drugs, because of the exponential increase in the number of possible switching directions (N = x!). The potential for stochastic orders to decelerate adaptation to antibiotic treatment is largely unexplored. Recent work demonstrated that stochastic sequences of three antibiotics can lead to very high treatment efficacy (i.e. high population extinction, low adaptation rate and reduced multidrug resistance [37]). Yet, not all stochastic sequential protocols produced similarly high efficacies [37].

**Box 1. The discovery of collateral sensitivity**

Collateral sensitivity is the specific term for trade-offs in antibiotic resistance, in which genetic changes that increase resistance to one antibiotic simultaneously increase susceptibility to other antibiotics. Collateral sensitivity was originally discovered and studied by Waclaw Szybalski at Cold Spring Harbor in the 1950s. Szybalski selected bacteria resistant to a wide array of antibiotics and toxic agents and screened them for cross-resistance against other antibiotics [50–52]. He discovered class-specific patterns in cross-resistance but also collateral sensitivity, and proposed to exploit these observations in chemotherapy [50]:

‘Whenever one antibiotic can be found that is particularly effective against bacteria resistant to another, it might be proved useful in combating disease and in permitting the application of antibiotics in a rational sequence when more than one is to be employed. Thus, the exact study of both collateral sensitivity and cross resistance may help in designing a proper program of multiple chemotherapy’.

However, at the time, antibiotic resistance was not common and research did not follow up on his ideas. Instead, his findings were mainly applied in the search for novel antibiotics [53]. Candidate substances were used to select for resistant mutants, which were screened for their collateral sensitivity profiles. A deviation of the mutant profiles from established profiles was taken as indication of a new class of antibiotic. In the following years, the term collateral sensitivity disappeared from the field of antibiotics research, although studies continued to accumulate evidence of sensitivity trade-offs in antibiotic resistance [26, 54, 55]. Only now—in the light of the antibiotic crisis—has this concept been re-connected to antibiotic therapy [18], and its applicability is currently being assessed. Matrices of evolved collateral effects have been inferred for *E. coli* and *P. aeruginosa* under laboratory conditions, and these studies simultaneously revealed the potential yet also the limitations of the concept. For example, collateral sensitivities involving aminoglycosides are very frequent, but their direction can vary among bacteria [18–21, 45] and between evolved replicates of the same strain [19, 46] depending on the precise genetic changes. Thus, the clinical exploitation of collateral sensitivity may be limited to cases of highly-predictable genetic interactions or depend on more precise diagnosis of the evolved collateral effects in the infecting population of pathogens.

**Box 2. Sequential application potentiates treatment due to physiological interactions**

Short exposures to sub-lethal antibiotic concentrations can potentiate subsequent antibiotic treatment. This phenomenon was first described in 1962 for *E. coli*. Pre-treatments of bacterial cultures with β-lactams for 15 min increased the bactericidal activity of aminoglycosides (AG, Figure panel A, modified from Ref. [33]) by accelerating their cellular uptake (Figure panel B, modified from Ref. [33]). Such physiological effects are likely important in a clinical study on a cohort of cystic fibrosis (CF) patients with chronic *P. aeruginosa* lung infections, published in 1988 and representing one of the very few clinical applications of fast sequential therapy (i.e. including drug changes within a patient in less than a day). This study evaluated the potency of a specific form of sequential treatment, where a second antibiotic is added while the first antibiotic was administered 4 h earlier. Physiological interactions should influence treatment outcome, even though they had not been known by the authors, because they switched between β-lactams and aminoglycosides, thus recapitulating the above described conditions. The test was unexpectedly successful, substantially reducing bacterial load upon sequential treatment (Figure panel C, modified from Ref. [15]):

‘Between 1983 and 1987, 36 episodes of pseudomonas infections in 32 patients with CF have been treated with a combination of a β-lactam (azlocillin, piperacillin, ticarcillin 120 mg/kg) and an aminoglycoside (gentamicin or tobramycin 12 mg/kg) with doses 4 h apart. In 16 episodes *P. aeruginosa* was eradicated from sputum for at least 3 weeks and sometimes for up to a year. In all other patients the number of colony forming units in sputum fell 1000-10 000-fold. Clinical improvement, as judged by fever, amount of sputum, and laboratory findings (e.g. erythrocyte sedimentation) was seen in every patient’. [15]

This strikingly contrasts with simultaneous dosing: ‘Between 1972 and 1978 we treated 66 episodes of infection due to *P. aeruginosa* in 52 patients with CF. We used a combination of carbenicillin (500 mg/kg) and an aminoglycoside (5 mg/kg) given simultaneously every 8 h. In none of these 66 episodes was the pathogen eradicated’ [15]. It is fascinating to see that this highly effective application of fast sequential therapy was not more widely explored.

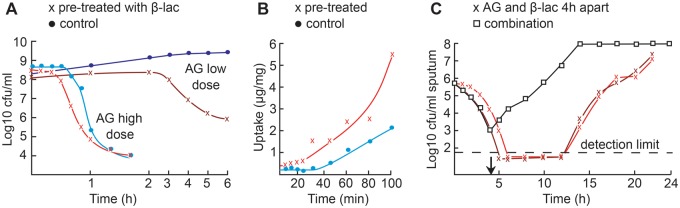

## FUTURE CHALLENGES

The consideration of principles from evolutionary ecology should help us refine antibiotic therapy, in order to reduce the rate of resistance emergence and spread. Fast-switching sequential treatments are a promising treatment alternative. Their particular potential is unfolded at the switches between antibiotics, because of the bactericidal effects of evolutionary and/or physiological trade-offs, such as collateral sensitivity and cellular hysteresis. Therefore, the key determinants for a successful application of sequential treatments are trade-off prevalence (and emergence), effect size and stability within the infecting population of the pathogen. As most work has been performed with laboratory strains of a few species, the prevalence of collateral sensitivity and negative hysteresis in clinical isolates has yet to be established. A recent study with a global collection of clinical *E. coli* isolates showed that collateral sensitivity is only mildly conserved [45]. An exception seems to be collateral sensitivity to aminoglycosides upon emergence of ciprofloxacin-resistance [45], which is also seen in *P. aeruginosa* [19, 22]. The emergence of collateral sensitivity (through evolution) is a probabilistic process [46], and the degree of predictability seems to be drug-dependent [19]. The application of collateral sensitivity may thus be limited to few conserved genetic interactions. Alternatively, it could be based on more detailed diagnosis of the evolved collateral effects in the infecting pathogen population, which however requires time and may thus only be useful for treating chronic or at least some type of long-lasting infection. Little is known as to the prevalence of negative hysteresis, although switches from β-lactam to aminoglycoside seems to be effective in *E. coli* [33]*, P. aeruginosa* [34, 37] and also resistant *P. aeruginosa* that overexpress the *mexAB-oprM* multidrug-efflux pump [37].

The success of fast sequential therapy also depends on the sustainability of the treatment benefit, and thus, it is inversely related to the ability of the bacteria to adapt to the imposed fluctuating environments. Sequential treatments with certain antibiotics lead to only small delays in resistance emergence [28, 40], and only switches between specific classes can cause re-sensitization to the earlier antibiotics [25, 28]. The conditions that determine the likelihood of re-sensitization have yet to be established. Even though sequential treatment restricts evolutionary potential, bacteria may ultimately be able to escape treatment constraints by rare evolutionary trajectories that lead to cross-resistance. The likelihood of cross-resistance then strongly depends on the choice of antibiotics. Ideally, the antibiotics select from distinct sets of beneficial mutations, which is often the case if they target different cellular functions, because cross-resistance is particularly common within drug classes. In general, we need more detailed information on how easily bacteria can evolve to break the exploited evolutionary or physiological trade-offs.

Furthermore, bacteria may adapt to unpredictable disturbances by increasing phenotypic heterogeneity, which can be produced by stochastic noise in gene expression [47]. The variability in gene expression contributes to antibiotic tolerance, due to growth rate dependent killing [48]. A certain frequency of nearly-dormant cells, so called persisters, is naturally produced by stochastic partitioning of proteins after cell division [49] and represents an ancient evolutionary survival strategy, bet-hedging, that can help bacterial populations to survive antibiotic exposure. Phenotypic heterogeneity may thus be an adaptive strategy for the bacteria to cope with unpredictable antibiotic treatments, thereby rendering them inefficient. To date, it is unclear to what extent such alternative life history strategies may emerge in response to sequential drug treatments. Moreover, availability of resistance-encoding plasmids may help the bacteria to escape evolved collateral effects or physiological constraints and thus generally the constraints by fluctuating selection conditions, because plasmid genes often show the potential for faster evolutionary change and more rapid spread within the bacterial population (in comparison to chromosomal genes). However, to date, the exact influence of such resistance-encoding plasmids on expression and stability of evolutionary or physiological trade-offs and also on sustainability of fast sequential therapy is as yet unexplored.

## CONCLUSIONS

In this commentary, we outlined how evolutionary principles can guide the development of novel antibiotic therapy. Previous work focussed on hospital-level approaches, that minimized transmission of resistance, and these studies showed that lowest overall resistance risk could be achieved by increasing temporal and spatial drug heterogeneity [4, 12]. We argue that these currently popular treatment designs are still sub-optimal, as they do not necessarily constrain bacterial adaptation within a patient. We here identify fast sequential therapy as a highly potent personalized treatment option that has the two-fold advantage of constraining resistance emergence and increasing bactericidal activity. Sequential therapy clearly warrants further exploration as a sustainable strategy to counter the antibiotic crisis. Bacterial evolution is highly dynamic. Why should our treatment designs remain as static as in Fleming’s time?
